# Parental Education Attainment and Educational Upward Mobility; Role of Race and Gender

**DOI:** 10.3390/bs8110107

**Published:** 2018-11-21

**Authors:** Shervin Assari

**Affiliations:** 1 Department of Psychiatry, University of Michigan, 4250 Plymouth Rd., Ann Arbor, MI 48109-2700, USA; assari@umich.edu; Tel.: +1-(310)-206-5162; 2 Center for Research on Ethnicity, Culture, and Health (CRECH), University of Michigan School of Public Health, Ann Arbor, MI 48109-2029, USA; 3 Department of Psychology, University of California Los Angeles (UCLA), Franz Hall, 502 Portola Plaza, Los Angeles, CA 90095, USA; 4 BRITE Center for Science, Research and Policy, University of California, Los Angeles (UCLA), Los Angeles, CA 90095, USA

**Keywords:** socioeconomic status (SES), socioeconomic mobility, educational mobility, social mobility, racism, African Americans, blacks

## Abstract

*Background.* The Minorities’ Diminished Return theory suggests that education attainment and other socioeconomic resources have smaller effects on the health and well-being of African Americans and other racial and ethnic minorities compared to Whites. Racial and ethnic differences in the processes involved with educational upward mobility may contribute to the diminished returns of education attainment for African Americans compared to Whites. Aim: This study compared African Americans and non-Hispanic Whites for the effect of parental education attainment on educational upward mobility and explored gender differences in these effects. *Methods.* The National Survey of American Life (NSAL 2003) is a nationally representative survey of American adults. Participants included 891 non-Hispanic White and 3570 African American adults. Gender, race/ethnicity, age, highest parental education attainment, and respondents’ educational attainment were measured. Data were analyzed using linear regression models. *Results.* Overall, higher parental education attainment was associated with higher educational upward mobility (b = 0.34, *p* < 0.001), however, this boosting effect was significantly smaller for African Americans compared to Whites (b = −0.13, *p* = 0.003). Our further analysis showed that race by parental education attainment can be found for females (b = −0.14, *p* = 0.013) but not males (*p* > 0.05). *Conclusion.* African American females are at a disadvantage compared to White females regarding the effect of parental education attainment on their educational upward mobility, a phenomenon which could not be observed when comparing African American and White males. These results advocate for taking intersectionality frameworks to study the effects of race, gender, and class in the US.

## 1. Introduction

Social and educational mobility has major implications for individuals’ health and well-being [1,2,3]. High socioeconomic status and access to more social resources reduce exposure to stress [4] and enhance one’s social network [5], health behaviors [3], materialistic and human resources [6,7], and access to social power [8,9]. Individuals and groups that are located at the top of the society face less risk factors and have better access to a wide range of social and economic buffers that can be relied on when the risk occurs [10]. High status SES boosts sense of control over life, self-efficacy, and sense of mastery [10] which are protective for the health and well-being of the individuals [11]. Through all these mechanisms, our relative position in the social hierarchy (in economic terms) has major implications for several health and well-being outcomes [12], as well explained in the “social gradient in health” [13] and “fundamental cause theory” [14,15].

Empirical evidence has documented subgroup (e.g., groups based on race, gender, and their intersections) variations in the effects of social status and socioeconomic and human resources on health [16,17,18]. This is in part because social mobility may entail differential processes for racial and race by gender groups [19,20]. According to the Minorities’ Diminished Return theory [16,17], upward social mobility as well as socioeconomic resources have smaller effects on the health and well-being of African Americans and other racial and ethnic minorities compared to Whites. Educational upward mobility differently influences the lives of Whites and African Americans, with social mobility altering exposure of Whites to social stress, however, for African Americans, exposure to stress is persistently high, regardless of intergenerational mobility [20]. For African Americans, high social status increases likelihood of contact with Whites at the workplace, neighborhood, and school, which in turn increases perceived discrimination [21,22,23,24]. As a result, the effects of education attainment of parent [25] and self [26] on stress [27], quality of life [28], well-being [29], subjective health [30], life satisfaction [31], mental distress [32,33], obesity [34,35,36], chronic disease [27], and mortality [37] are systemically smaller for African Americans in comparison to Whites [38,39].

The processes and outcomes of social and educational mobility, however, are not exclusively shaped by race as they are also influenced by other social identities such as gender [18,19]. In a study on a national sample of African Americans, education attainment showed a protective effect against psychological distress for females but not males [18]. In another study by Chetty et al., [19] it was not race/ethnicity but the intersection of race/ethnicity and gender that altered likelihood of social mobility in the US. In their study, African American males were the group with least likelihood to be upwardly mobile and most likely to experience downward social mobility [19]. This is probably because racism does differently block chances for upward social mobility of African American males and females with African American males at a relative disadvantage in comparison to African American females when it comes to racism and discrimination [40]. This is also supported by the considerable evidence suggesting that high SES (e.g., high education attainment and income) operate as a vulnerability factor for African American males but not females [21,22,41,42,43].

### Aims

The current study used a nationally representative sample of American adults to investigate the effects of race/ethnicity and parental education attainment on intergenerational educational mobility (net algebraic difference between parent and own education attainment) of Americans. We are particularly interested in the multiplicative rather than additive effects of race/ethnicity and parental education attainment on own education attainment (intergenerational educational mobility). We also explored gender differences in these effects.

We argue that the processes involved with intergenerational upward educational mobility are not universal but specific to each racial and ethnic group. At the same time, SES and social mobility have different implications for the well-being of diverse groups. We specifically hypothesize that intergenerational educational mobility is not the result of algebraic sum of race/ethnicity and resources (e.g., parental education) but their interactive and multiplicative effects. Although we expect parent education to boost respondent education attainment of the offspring, we expect this boosting effect to be smaller for African Americans than Whites, in line with the Minorities’ Diminished Returns [16,17] and also the empirical evidence on transgenerational transition of human and materialistic resources [44,45]. Guided by the results by Chetty et al., [19] and other research [18], we also expect considerable gender differences in these effects. To be more specific, we expect race by parent education interaction for African American males but not females [19].

The results are expected to help us with understanding why social mobility, social status, and SES resources are far less protective for African Americans compared to Whites [46,47,48,49], as described in the Minorities’ Diminished Returns theory [16,17]. The results may also shed light on why upward educational mobility and high education attainment may increase vulnerability of African Americans, particularly men [43].

## 2. Methods

### 2.1. Design and Settings

The National Survey of American Life (NSAL) is a landmark cross-sectional mental health survey of African American adults and a non-Hispanic White control group in the US [50,51,52]. We briefly describe the study design and methods, however, more details on this regard are available elsewhere [50,51,52].

### 2.2. Participants and Sampling

The NSAL study used a household probability sample to generate a nationally representative sample of American adults. In the NSAL study, African Americans and non-Hispanic Whites were drawn from rural areas, large cities, and urban areas [50,51,52]. Using a multistage sampling design, the NSAL enrolled African Americans and Whites if they were (1) adults (age 18 years and older), (2) resided in the coterminous US (i.e., 48 states), (3) noninstitutionalized individuals, and (4) able to conduct a structured interview in English. Exclusion criteria included residing in nursing homes, long-term medical care settings, prisons, and jails or not speaking English [50,51,52]. The analytical sample for this paper included 4461 adults (891 non-Hispanic Whites and 3570 African Americans).

### 2.3. Data Collection and Procesures

Data were collected using structured interviews. All interviews were conducted in English. The interviewee and interviewers were matched on race. Most (82%) of all NSAL interviews were conducted as face-to-face and only 14% of them were conducted by telephone. Computer-Assisted Personal Interviewing (CAPI) was used for the face-to-face interviews. In CAPI, computers are used to assist participants answer lengthy questionnaires with complex skip patterns. Some evidence suggests that CAPI enhances data quality particularly for long and complex surveys [53]. Interviews took 140 min on average to complete. The response rate was 71% and 70% for African Americans and non-Hispanic Whites, respectively.

### 2.4. Measures and Variables

The study constructs used for the current analysis included race/ethnicity (measured), gender (measured), age (measured), parental education attainment (measured), respondent’s education attainment (measured), and intergenerational upward educational mobility (calculated).

*Race/Ethnicity.* Race/ethnicity was measured as self-identified. Participants self-reported their race either as African American or White Americans. African Americans were defined as Blacks who do not have ancestral ties to any Caribbean countries.

*Education Attainment.* Years of schooling (education) were measured using self-reported data. This variable was treated as a continuous measure which could potentially range from 4 to 17. Separate variables measured maternal education attainment, paternal education attainment, and respondent’s education attainment, all being self-reported. That is, participants were asked to report the number of years of schooling of their own and their parents’ including their mothers (or the women who raised them) and fathers (or the men who raised them). In cases where education information was only available for one parent, that parent’s education attainment was considered as highest parental education. When education information was reported for both parents, the highest level of education was considered as parental educational attainment.

*Intergenerational Educational Upward Mobility.* Intergenerational educational mobility was conceptualized as the algebraic difference between respondent’s and their parents’ years of education. A positive score was indicative of an upward intergenerational educational mobility (individual who have higher education attainment than their parents), a score of zero was indicative of no intergenerational educational mobility (respondents with similar education attainment compared to their parents), and a negative score was indicative of a downward intergenerational educational mobility (respondents who have less education than their parents). This variable was operationalized as an interval variable [54].

### 2.5. Statistical Analysis

Given the complex sampling design of the NSAL, we used sampling weights for data analysis. We used Stata 15.0 (Stata Corp; College Station, TX, USA) for univariate, bivariate, and multivariable data analysis. We used Taylor series linearization for recalculation of the design-based standard errors (SEs), thus all the inferences reported here are representative to the US adult population. Given that we were only interested in comparing Whites and African Americans, we used sub-pop survey commands. We performed all of our analyses for the pooled sample as well as specific to each race/ethnic group.

For descriptive purposes, we used survey mean and proportions (%). For bivariate analysis that compares Whites and African Americans, we applied independent samples test as well as Pearson Chi-square test. For multivariable analysis, we used four linear regression models. From our regression analyses, we reported unstandardized (adjusted) regression coefficients (b), 95% confidence intervals (CIs), z, and *p* levels.

Four linear regression models were fitted to the data. To operationalize intergenerational educational mobility as the outcome, we considered parental education attainment as the independent variable and own education attainment as the dependent variable. Due to this operationalization, outcome is in fact the own education after controlling for parental education, which is intergenerational educational mobility. Age was the covariate. Race/ethnicity and gender were the focal moderators. The first two linear regression models were estimated in the pooled sample that included both Whites and African Americans. Model 1-a did not include any interaction term. Model 2-a also included a race by parental education interaction term. Model 3-a and Model 4-a were conducted for each racial/ethnic group. That is Model 3-a for Whites and Model 4-a for African Americans only. We also ran similar models for males (Model 1-b to Model 4-b) and females (Model 1-c to Model 4-c).

## 3. Results

### 3.1. Descriptive Statistics

This study included a total number of 4461 American adults who were non-Hispanic Whites (*n* = 891) or African Americans (*n* = 3570). Table 1 provides the summary of the descriptive statistics in the pooled sample and by race/ethnicity. African Americans had lower parental and own education attainment compared to Whites, however, they had higher intergenerational educational mobility than Whites (*p* < 0.05). African Americans and Whites also differed in age and gender, with African Americans being younger and being more composed of women, compared to Whites.

### 3.2. Linear Regressions for Both Genders

Table 2 summarizes the results of four linear regression models with parental educational attainment as the main predictor of interest and own education attainment as the main outcome. Model 1-a and Model 2-a were in the pooled sample. Model 3-a and Model 4-a were for White Americans and for African Americans. Model 1-a, which only included the main effects, showed a significant positive association between parental education attainment on intergenerational educational mobility, net of race, age, and gender. Model 2-a showed a significant interaction between race/ethnicity and parental education on intergenerational educational mobility, showing a smaller effect of parental education on intergenerational educational mobility for African Americans compared to Whites.

### 3.3. Linear Regressions for Males

Table 3 summarizes the results of four other linear regression models for males (Model 1-b to Model 4-b). The interaction between race and parental education attainment could not be found for males.

### 3.4. Linear Regressions for Females

Table 4 summarizes of the results of four other linear regression models for females (Model 1-c to Model 4-c). Our further analysis showed that the interaction between race and parental education attainment could be found for females.

## 4. Discussion

Using a nationally representative sample of White and African American adults, the current study showed three findings. First, overall, race was not associated with intergenerational educational mobility (i.e., the difference between respondent’s and parents’ education attainment). Second, having higher parental education attainment was positively associated with intergenerational educational mobility. Third, the effect of highest parental education attainment on respondent education attainment was larger for Whites than African Americans. Forth, this race difference (smaller effect of parental education attainment on intergenerational upward educational mobility) could be only found for females but not males.

Race did not have a main effect on the intergenerational educational mobility in our multivariable analysis, however, our bivariate analysis showed a larger net difference between respondents’ and parents’ education for African Americans comparison to Whites. The bivariate effect may be due to ceiling effects for Whites/floor effects for Blacks [55,56], given that in previous generations, African Americans had disproportionately lower education attainment than Whites [57,58]. The historical gap in education attainment of races would mean more room for improvement for Blacks than Whites. Filling such historical cap would require multiple generations to narrow and close. As a result, we expected more improvement for the new generations of African Americans to surpass education attainment of their parents, compared to Whites. Although civil right movement enhanced African Americans’ education opportunities [59], such social changes occur with some considerable time lag, which may be decades and generations [60]. Race not impacting intergenerational educational mobility in US suggests that the historical gap in education attainment between African Americans and Whites is not narrowing rapidly.

Parental education attainment boosted the educational mobility of the offspring. This effect is probably due to the positive effect of parental education on the academic success of their children [61,62,63] as well as the education orientated mind set and value system of families in whom parents are educated [64,65,66]. A considerable education literature has well-established the role of parental education attainment as a robust driver of academic success and attainment of the children [61,62,63]. It is considerably more difficult to attain high education attainment for children if their parents are not able to educationally support them, probably due to their low education [67].

We found smaller effects of parental education attainment on educational mobility of offspring for African Americans compared to Whites, which is in line with the Minorities’ Diminished Return theory [16,17,38,39]. A number of intergenerational and cross-generational studies have documented similar racial differences in the returns of parental education on child outcomes. In the National Survey of Children’s Health (NSCH) 2003–2004 that included 86,537 families with children 0–17 years old, major racial differences were found in the effects of parental education on family’s poverty risk. Although overall, higher parental education was associated with lower risk of poverty; this effect was considerably smaller for African American compared to White families [48]. The second study used the MIDUS data, with 10 years of follow-up and showed that education attainment at baseline predicted future increase in income for White but not African American families [68]. Three other studies used 15 years follow-up data of 1781 families from birth of their child to age 15 in the Fragile Families and Child Wellbeing Study (FFCWS) [44,45,69]. Although overall, maternal education at birth was protective against obesity [44], poor mental health [69], and impulsivity [45] of youth at 15 years of age; all these effects were systemically smaller for African American than White families. In one FFCWS, the same education level of parents had a larger effect on the child GPA than African American families [70]. In all these studies, race has interacted with maternal education at birth on future youth outcome, and African American families have always gained less than White families, regardless of the type of outcome. These findings are explained as the Diminished Returns of Minorities [16,17].

Thus, it is not “race or class” but “race and class” that generate racial inequalities in the US [71]. This argument is well explained by Williams [72,73,74], Navarro [71], Shapiro [75], and Ferraro [38] among others. Racial disparities persist even at the top of the society (highest SES levels) [76,39]. As a result, eliminating racial differences in class and SES will not be enough for elimination of the racial differences in outcomes. Policies that merely address equal opportunity will not result in equal outcomes across racial groups [16,17].

In the current political climate where racism, White supremacy, and explicit racism is back in the everyday life of Americans, race and ethnic minority groups do not have equal chance for upward social mobility in the US. Even after successfully climbing the social ladder, subgroups of the society differ in how much tangible gains they receive from their parental and their own SES resources. As shown in this study, maternal education is one of the human resources that fails to generate equal tangible outcomes (respondent’s education attainment) for African Americans and Whites [44,45,48,49,69].

Upward social mobility and high SES bring smaller changes to the lives of African Americans as well as other race and ethnic minority groups who have lived a life full of oppression and adversity. At each level of SES, African Americans’ lives are not comparable to those of Whites, most of whom live a privileged life [16,17]. As race/ethnicity is a proxy of how a group and an individual is treated by the society, and race (i.e., skin color) shapes our access to the opportunity structure [77,78], African Americans still need to fight an uphill battle, as their social mobility cannot “buy them Whiteness” [79]. As a result, despite successfully climbing the social ladder, they still gain far less than their White Americans.

The group differences observed in this study are not likely attributable to the different choices that different groups make, but the different treatment that groups receive by the society. As a result of unfair social encounters, African Americans face many constrains in their access to the opportunity structure and social power due to pervasive structural and institutional racism. Across domains and at each level of SES, society has a preference toward Whites to non-Whites, thus upwardly social mobile African Americans will still face unfair treatment by the society, which results in SES consistently generate diminished returns for non-Whites [38]. The relative disadvantage of African Americans to Whites is largely due to racism and discrimination [21,22,43] that are rampant across levels and institutions.

These group differences cannot be reduced to cultural differences (e.g., culture of poverty) [80]. Instead of finding a solution, society should stop blaming the victims (e.g., African Americans) [81]. In the same lines, these differences should not be interpreted as innate group differences in intelligence or that one group is more efficient in translating their resources to outcomes than other [82]. Even if racial and ethnic minority groups have a higher tendency to use psychologically taxing coping styles, the root cause is not their maladaptive coping but the rampant stress due to persistent racism [83,84,85].

Although residential segregation and education quality can explain diminished returns of parental education on offspring education in African Americans than White Americans, however, they cannot explain the observed gender differences in the relevance of race and parental education on upward social mobility of African Americans [86]. Due to residential segregation, opportunities are scarcer in urban places where African Americans live compared to suburban areas where Whites predominantly reside [87,88,89]. Lower education quality and resources in predominantly African American communities adversely impact children educational success even when parents are motivated and educated [90,91]. In addition, highly educated African American families stay at higher risk of poverty compared to their White counterparts [48], which means survival needs may become priority and education of the offspring may become secondary. Several other economic, societal, and psychological processes such as stressful life events [20], discrimination [43], and social mobility stress [92] can explain the diminished returns of parental education attainment for African American families relative to Whites. For example, due to structural racism, the labor market [93] and education system [48,49] treat African Americans unfairly, thus education attainment generates smaller tangible outcomes for African Americans than Whites [16,17].

There are also some sociological processes and psychological mechanisms (e.g., structural racism and interpersonal discrimination) that may result in gender differences in the effects of race and racism [21,22,43,55]. That means, some of the processes involved in racism may differ for males and females. For instance, discrimination by the education system [94], labor market [95,96,97,98,99], police, and correctional setting [100,101,102] may be worse for males than females. These may explain why experience and vulnerability to discrimination is worse for African American males than females [40], why high SES increases vulnerability to discrimination [103], why education attainment protects female but not male African Americans [18], and why high SES African American men and boys but not women and girls are more depressed [42,83,104,105,106,107]. Understanding race by gender variation in these processes are essential for an understanding of the diminished returns of African American men [43].

The results help us understand why parent education does not generate as many health benefits for African American offspring [44,45,69], and why SES fails to reduce poor mental health [59], discrimination [91], stress [20], and SES-related stress [92] for African Americans as Whites. Less information is available on why mental health particularly depression is worse among high SES African Americans, particularly for high SES African American males [42,83,104,105,106,107]. All this information helps us understand the results by Steele [108], Hudson [106,107,109], and Fuller-Rowell [110] who have shown smaller health gain from upward social mobility in African Americans than Whites. The effects of education attainment of parent [25] and self [26] on stress [27], quality of life [28], well-being [29], subjective health [30], life satisfaction [31], mental distress [32], obesity [34,35,36], chronic disease [27], and mortality [37] are smaller for African Americans relative to Whites, as explained by the Minorities’ Diminished Return theory [16,17,38,39].

The current results extend the exiting literature on the intersections of race/ethnicity, gender/sex, and SES/class on mental health. The results advocate for taking an intersectionality approach to study the effects of race, gender, and class [111,112,113,114,115]. Using this framework, the profile and situation of each intersectionality group is not the algebraic sum of the identities that make the intersection, but the multiplicative effects of such social identities.

### 4.1. Study Limitations

Our study is not without limitations. First, due to cross-sectional design, the current results are indicative of association rather than causation. Thus by the term effect, we refer to statistical rather than causal effects. While race and parental education are likely to impact offspring educational outcomes, reverse causality is not likely. Still, future research should use a longitudinal design and follow families and individuals over time and use repeated observations of education, health, and well-being. Second, the current study controlled for a limited number of confounders. More research is needed while a more inclusive list of covariates are included (i.e., childhood SES, area level SES, and contextual factors such as racial composition). Third, this study relied on self-report of parental education which is prone to measurement bias. Forth, the data were old, as the NSAL was conducted between 2001 and 2003. Despite these methodological limitations, this study contributes to the existing knowledge on racial differences in the process of intergenerational social mobility [42,77,78,84,116,117].

### 4.2. Future Research

The existing knowledge is very limited regarding how the intersections of race/ethnicity, class, and gender shape inequalities and disparities, and how the effects of one’s social identity (e.g., race) depend on other social identities (e.g., gender). By taking an intersectionality approach, future research should study the nonlinear nature of interactions between various social identities that jointly operate and shape the health and well-being of subgroups of populations. To date, most of the research has focused on separate rather than combined and linear rather than nonlinear effects of race/ethnicity, gender, and class. In addition, more focus is given to lack of resources (e.g., poverty) rather than diminished return of available resources (e.g., educated and middle class families). It is also unknown what role contextual and individual factors such as discrimination, stress, segregation, neighborhood resources, labor market preferences, and poor quality of schooling in urban areas play in explaining the differences between African Americans and Whites in upward social mobility and gaining health from it. It is also not clear what role early exposure during childhood plays and what percentage of these racial heterogeneities are due to the disadvantage that accumulates over the life course. Future research may also define social mobility in terms of occupation, income, and wealth. Finally, more research is needed across cohorts and age groups on differences between Whites and African Americans in gaining tangible outcomes from upward social mobility. There is a need for test of replication of our results using more recent data, particularly on other markers of class and social mobility (e.g., income, wealth, and occupation).

## 5. Conclusions

To conclude, our findings suggest that the effect of parental education attainment on intergenerational educational upward mobility is diminished for African American females compared to White females. As the effect of racism on social mobility is not the same for males and females, we advocate for application of an intersectionality approach for future research on race, gender, and social mobility/class.

### Ethics

The University of Michigan (UM) Institute Review Board (IRB) approved the NSAL study protocol. Written informed consent was received from all the participants. Respondents received financial compensation for their time.

## Figures and Tables

**Table 1:** Descriptive statistics in the pooled sample and by race/ethnicity.

	All (*n* = 4461)		African American (*n* = 891)		Non-Hispanic White (*n* = 891)	
	% (SE)	95% CI	% (SE)	95% CI	% (SE)	95% CI
Gender						
Male	45.69 (0.01)	43.43–47.97	44.03 (0.01)	42.35–45.72	47.26 (0.02)	42.89–51.66
Female	54.31 (0.01)	52.03–56.57	55.97 (0.01) *	54.28–57.65	52.74 (0.02)	48.34–57.11
	**Mean (SE)**	**95% CI**	**Mean (SE)**	**95% CI**	**Mean (SE)**	**95% CI**
Age	43.54 (0.71)	42.11–44.97	42.07(0.53)	40.98–43.16	44.90 (1.31) *	42.10–47.70
Parental Education (Years)	12.08 (0.18)	11.71–12.44	11.30(0.11)	11.08–11.53	12.75 (0.29) *	12.13–13.36
Own Education (Years)	13.13 (0.15)	12.83–13.43	12.69 (0.09)	12.51–12.87	13.51 (0.27) *	12.94–14.08
Educational Mobility (Years)	1.06 (0.13)	0.81–1.31	1.39 (0.08) *	1.22–1.56	0.76 (0.21)	0.31–1.22

Notes: Standard Error (SE), Confidence Interval (CI), * *p* < 0.05.

**Table 2:** Linear regressions between race, parental education attainment, and upward educational mobility.

	b(SE)	95% CI	t	*p*
***Model 1-a***				
Race (African Americans)	−0.29(0.22)	−0.72–0.15	−1.32	0.192
Gender (Female)	−0.16(0.15)	−0.47–0.15	−1.05	0.299
Parental Education (Years)	0.34(0.02)	0.30–0.39	16.13	0.000
Age	0.01(0.00)	0.01–0.02	3.28	0.002
Intercept	8.57(0.44)	7.69–9.45	19.61	0.000
***Model 2-a (Model 1-a + Interactions)***				
Race (African Americans)	1.25(0.46)	0.31–2.18	2.68	0.010
Gender (Female)	−0.15(0.16)	−0.46–0.17	−0.94	0.351
Parental Education (Years)	0.41(0.04)	0.33–0.48	11.00	0.000
Age	0.01(0.00)	0.01–0.02	3.36	0.002
Race × Parental Education (Years)	−0.13(0.04)	−0.21–−0.05	−3.18	0.003
Intercept	7.76(0.54)	6.67–8.85	14.30	0.000
***Model 3-a (White Americans)***				
Gender (Female)	−0.19(0.29)	−0.79–0.42	−0.65	0.523
Age	0.40(0.04)	0.32–0.48	10.29	0.000
Parental Education (Years)	0.01(0.01)	0.00–0.03	1.73	0.103
Intercept	7.95(0.71)	6.44–9.46	11.20	0.000
***Model 4-a (African Americans)***				
Gender (Female)	−0.10(0.09)	−0.29–0.09	−1.08	0.286
Age	0.29(0.02)	0.25–0.32	15.06	0.000
Parental Education (Years)	0.02(0.00)	0.01–0.03	4.05	0.000
Intercept	8.77(0.34)	8.07–9.47	25.46	0.000

Outcome: Own Education Attainment.

**Table 3:** Linear regressions between race, parental education attainment, and upward educational mobility in males.

	b(SE)	95% CI	t	*p*
***Model 1-c***				
Race (African Americans)	−0.24(0.29)	−0.82–0.35	−0.81	0.420
Parental Education (Years)	0.34(0.04)	0.26–0.43	8.16	0.000
Age	0.02(0.01)	0.00–0.03	2.36	0.023
Intercept	8.34(0.64)	7.06–9.63	13.03	0.000
***Model 2-c (Model 1-c + Interactions)***				
Race (African Americans)	0.01(0.01)	0.00–0.03	2.26	0.028
Parental Education (Years)	0.35(0.05)	0.26–0.44	7.72	0.000
Age	0.00 (-)			
Race × Parental Education (Years)	−0.03(0.02)	−0.07–0.02	−1.13	0.262
Intercept	8.30(0.61)	7.08–9.52	13.66	0.000
***Model 3-c (White Americans)***				
Parental Education (Years)	0.40(0.07)	0.25–0.55	5.61	0.000
Age	0.02(0.01)	−0.01–0.04	1.48	0.158
Intercept	7.62(1.08)	5.32–9.91	7.08	0.000
***Model 4-c (African Americans)***				
Parental Education (Years)	0.28(0.03)	0.21–0.35	8.17	0.000
Age	0.01(0.01)	0.00–0.03	2.73	0.010
Intercept	8.89(0.01)	7.87–9.92	17.57	0.000

Outcome: Own Education Attainment.

**Table 4:** Linear regressions between race, parental education attainment, and upward educational mobility in females.

	b(SE)	95% CI	t	*p*
***Model 1-b***				
Race (African Americans)	−0.33(0.21)	−0.75–0.10	−1.55	0.128
Age	0.34(0.03)	0.29–0.40	12.56	0.000
Parental Education (Years)	0.01(0.00)	0.01–0.02	3.22	0.002
Intercept	8.62(0.46)	7.69–9.56	18.55	0.000
***Model 2-b (Model 1-b + Interactions)***				
Race (African Americans)	1.35(0.62)	0.11–2.60	2.19	0.034
Age	0.42(0.05)	0.31–0.52	8.18	0.000
Parental Education (Years)	0.01(0.00)	0.01–0.02	3.36	0.002
Race × Parental Education (Years)	−0.14(0.05)	−0.25–−0.03	−2.58	0.013
Intercept	7.66(0.65)	6.35–8.97	11.74	0.000
***Model 3-b (White Americans)***				
Age	0.41(0.05)	0.30–0.52	7.82	0.000
Parental Education (Years)	0.01(0.01)	0.00–0.02	1.48	0.159
Intercept	8.00(0.74)	6.42–9.57	10.83	0.000
***Model 4-b (African Americans)***				
Age	0.29(0.02)	0.24–0.34	12.41	0.000
Parental Education (Years)	0.02(0.01)	0.01–0.03	3.52	0.001
Intercept	8.60(0.46)	7.67–9.52	18.86	0.000

Outcome: Own Education Attainment.

## References

[1] Rahkonen O., Arber S., Lahelma E. (1997). Health-related social mobility: A comparison of currently employed men and women in Britain and Finland. Scand. J. Soc. Med..

[2] Hart C.L., Smith G.D., Blane D. (1998). Social mobility and 21 year mortality in a cohort of Scottish men. Soc. Sci. Med..

[3] Letelier A., Heilmann A., Watt R.G., Jivraj S., Tsakos G. (2016). Does intergenerational social mobility affect the general health, oral health, and physical function of older adults in England?. Lancet.

[4] Aneshensel C.S. (1992). Social stress: Theory and research. Annu. Rev. Soc..

[5] Podolny J.M., Baron J.N. (1997). Resources and relationships: Social networks and mobility in the workplace. Am. Soc. Rev..

[6] Coleman J.S. (1988). Social capital in the creation of human capital. Am. J. Soc..

[7] Marsden P.V., Hurlbert J.S. (1988). Social resources and mobility outcomes: A replication and extension. Soc. Forces.

[8] Hollingshead A.B. (1975). Four Factor Index of Social Status.

[9] Wegener B. (1991). Job mobility and social ties: Social resources, prior job, and status attainment. Am. Soc. Rev..

[10] Marmot M.G., Bosma H., Hemingway H., Brunner E., Stansfeld S. (1997). Contribution of job control and other risk factors to social variations in coronary heart disease incidence. Lancet.

[11] Surtees P.G., Wainwright N.W., Luben R., Khaw K.T., Day N.E. (2006). Mastery, sense of coherence, and mortality: Evidence of independent associations from the EPIC-Norfolk Prospective Cohort Study. Health Psychol..

[12] Blane D., Harding S., Rosato M. (1999). Does social mobility affect the size of the socioeconomic mortality differential?: Evidence from the Office for National Statistics Longitudinal Study. J. R. Stat. Soc. Ser. A.

[13] Marmot M. (2005). Social determinants of health inequalities. Lancet.

[14] Link B.G., Phelan J. (1995). Social conditions as fundamental causes of disease. J. Health Soc. Behav..

[15] Phelan J.C., Link B.G. (2013). Fundamental cause theory. Medical Sociology on the Move.

[16] Assari S. (2018). Unequal gain of equal resources across racial groups. Int. J. Health Policy Manag..

[17] Assari S. (2018). Health disparities due to blacks’ diminished return: Public Policy Solutions. Soc. Issues Policy Rev..

[18] Assari S. (2018). Educational Attainment Better Protects African American Women than African American Men Against Depressive Symptoms and Psychological Distress. Brain Sci..

[19] Chetty R., Hendren N., Jones M.R., Porter S.R. (2018). Race and Economic Opportunity in the United States: An Intergenerational Perspective. NBER Working Paper No. 24441. http://www.nber.org/papers/w24441.

[20] Assari S. (2018). Race, Intergenerational Social Mobility and Stressful Life Events. Behav. Sci..

[21] Assari S., Gibbons F., Simons R. (2018). Depression among Black youth; Interaction between Place and Class. Brain Sci..

[22] Assari S., Gibbons F.X., Simons R. (2018). Social Determinants of perceived Discrimination among Black youth in the United States, an 18-Year Longitudinal Study. Behav. Sci..

[23] Assari S. (2018). Does School Racial Composition Explain Why High Income Black Youth Perceive More Discrimination? A Gender Analysis. Brain Sci..

[24] Assari S., Lankarani M.M. (2018). Workplace Racial Composition Explains High Perceived Discrimination of High Socioeconomic Status African American Men. Brain Sci..

[25] Carozza S.E., Puumala S.E., Chow E.J., Fox E.E., Horel S., Johnson K.J., McLaughlin C.C., Reynolds P., von Behren J., Mueller B.A. (2010). Parental educational attainment as an indicator of socioeconomic status and risk of childhood cancers. Br. J. Cancer.

[26] Lubetkin E.I., Jia H., Franks P., Gold M.R. (2005). Relationship among sociodemographic factors, clinical conditions, and health-related quality of life: Examining the EQ-5D in the US general population. Qual. Life Res..

[27] Baum A., Garofalo J.P., Yali A.M. (1999). Socioeconomic status and chronic stress: Does stress account for SES effects on health?. Ann. N. Y. Acad. Sci..

[28] Apter A.J., Reisine S.T., Affleck G., Barrows E., ZuWallack R.L. (1999). The influence of demographic and socioeconomic factors on health-related quality of life in asthma. J. Allergy Clin. Immunol..

[29] Colet C.D.F., Mayorga P., Amador T.A. (2010). Educational level, socio-economic status and relationship with quality of life in elderly residents of the city of Porto Alegre/RS, Brazil. Braz. J. Pharm. Sci..

[30] Alvarez-Galvez J., Rodero-Cosano M.L., Motrico E., Salinas-Perez J.A., Garcia-Alonso C., Salvador-Carulla L. (2013). The impact of socio-economic status on self-rated health: Study of 29 countries using European social surveys (2002–2008). Int. J. Environ. Res. Public Health.

[31] Bjørnskov C. (2003). The happy few: Cross–country evidence on social capital and life satisfaction. Kyklos.

[32] Phongsavan P., Chey T., Bauman A., Brooks R., Silove D. (2006). Social capital, socio-economic status and psychological distress among Australian adults. Soc. Sci. Med..

[33] Assari S. (2018). Parental Educational Attainment and Mental Well-Being of College Students; Diminished Returns of Blacks. Brain Sci..

[34] Sobal J., Stunkard A.J. (1989). Socioeconomic status and obesity: A review of the literature. Psychol. Bull..

[35] Ogden C.L., Lamb M.M., Carroll M.D., Flegal K.M. (2010). Obesity and Socioeconomic Status in Children and Adolescents: United States, 2005–2008. NCHS Data Brief. Number 51.

[36] Winkleby M.A., Jatulis D.E., Frank E., Fortmann S.P. (1992). Socioeconomic status and health: How education, income, and occupation contribute to risk factors for cardiovascular disease. Am. J. Public Health.

[37] Everett B.G., Rehkopf D.H., Rogers R.G. (2013). The nonlinear relationship between education and mortality: An examination of cohort, race/ethnic, and gender differences. Popul. Res. Policy Rev..

[38] Farmer M.M., Ferraro K.F. (2005). Are racial disparities in health conditional on socioeconomic status?. Soc. Sci. Med..

[39] Hastert T.A. (2017). All dollars are not created equal: Health disparities persist even among the highest income Americans. Prev. Med..

[40] Assari S., Moazen-Zadeh E., Caldwell C.H., Zimmerman M.A. (2017). Racial Discrimination during Adolescence Predicts Mental Health Deterioration in Adulthood: Gender Differences among Blacks. Front. Public Health.

[41] Assari S. (2017). Social Determinants of Depression: The Intersections of Race, Gender, and Socioeconomic Status. Brain Sci..

[42] Assari S., Caldwell C.H. (2017). High Risk of Depression in High-Income African American Boys. J. Racial Ethn. Health Disparities.

[43] Assari S., Lankarani M.M., Caldwell C.H. (2018). Does Discrimination Explain High Risk of Depression among High-Income African American Men?. Behav. Sci..

[44] Assari S., Thomas A., Caldwell C.H., Mincy R.B. (2018). Blacks’ Diminished Health Return of Family Structure and Socioeconomic Status; 15 Years of Follow-up of a National Urban Sample of Youth. J. Urban Health.

[45] Assari S., Caldwell C.H., Mincy R. (2018). Family Socioeconomic Status at Birth and Youth Impulsivity at Age 15; Blacks’ Diminished Return. Children.

[46] Assari S. (2018). The Benefits of Higher Income in Protecting against Chronic Medical Conditions Are Smaller for African Americans than Whites. Healthcare.

[47] Assari S. (2018). Family Income reduces risk of childhood obesity for Whites but not Blacks. Children.

[48] Assari S. (2018). Parental Education Better Helps White than Black Families Escape Poverty: National Survey of Children’s Health. Economies.

[49] Assari S. (2018). Diminished Economic Return of Socioeconomic Status for Black Families. Soc. Sci..

[50] Heeringa S.G., Wagner J., Torres M., Duan N., Adams T., Berglund P. (2004). Sample designs and sampling methods for the Collaborative Psychiatric Epidemiology Studies (CPES). Int. J. Methods Psychiatr. Res..

[51] Jackson J.S., Neighbors H.W., Nesse R.M., Trierweiler S.J., Torres M. (2004). Methodological innovations in the National Survey of American Life. Int. J. Methods Psychiatr. Res..

[52] Jackson J.S., Torres M., Caldwell C.H., Neighbors H.W., Nesse R.M., Taylor R.J., Trierweiler S.J., Williams D.R. (2004). The National Survey of American Life: A study of racial, ethnic and cultural influences on mental disorders and mental health. Int. J. Methods Psychiatr. Res..

[53] Birkett N.J. (1988). Computer-aided personal interviewing. A new technique for data collection in epidemiologic surveys. Am. J. Epidemiol..

[54] Mouzon D.M., Watkins D.C., Perry R., Simpson T.M., Mitchell J.A. (2018). Intergenerational Mobility and Goal-Striving Stress Among Black Americans: The Roles of Ethnicity and Nativity Status. J. Immigr. Minor. Health.

[55] Bindman A.B. (1990). Measuring health changes among severely ill patients. The floor phenomenon. Med. Care.

[56] Dean K., Walker Z., Jenkinson C. (2018). Data quality, floor and ceiling effects, and test-retest reliability of the Mild Cognitive Impairment Questionnaire. Patient Relat. Outcome Meas..

[57] Darling-Hammond L. (1998). Unequal opportunity: Race and education. Brook. Rev..

[58] Tate W.F. (1997). Chapter 4: Critical race theory and education: History, theory, and implications. Rev. Res. Educ..

[59] Donohue J.J., Heckman J. (1991). Continuous Versus Episodic Change: The Impact of Civil Rights Policy on the Economic Status of Blacks.

[60] DiPrete T.A., Buchmann C. (2006). Gender-specific trends in the value of education and the emerging gender gap in college completion. Demography.

[61] Dubow E.F., Boxer P., Huesmann L.R. (2009). Long-term Effects of Parents’ Education on Children’s Educational and Occupational Success: Mediation by Family Interactions, Child Aggression, and Teenage Aspirations. Merrill Palmer Q..

[62] Dubow E.F., Huesmann L.R., Boxer P., Pulkkinen L., Kokko K. (2006). Middle childhood and adolescent contextual and personal predictors of adult educational and occupational outcomes: A mediational model in two countries. Dev. Psychol..

[63] Dubow E.F., Huesmann L.R., Boxer P., Smith C. (2014). Childhood predictors and age 48 outcomes of self-reports and official records of offending. Crim. Behav. Ment. Health.

[64] DeCastro-Ambrosetti D., Cho G. (2005). Do Parents Value Education? Teachers’ Perceptions of Minority Parents. Multicult. Educ..

[65] Jacob B.A., Lefgren L. (2007). What do parents value in education? An empirical investigation of parents’ revealed preferences for teachers. Q. J. Econ..

[66] Valencia R.R. (2002). Mexican Americans don’t value education!” On the basis of the myth, mythmaking, and debunking. J. Lat. Educ..

[67] Choy S. (2001). Students Whose Parents Did Not Go to College: Postsecondary Access, Persistence, and Attainment. Findings from the Condition of Education, 2001.

[68] Assari S., Preiser B., Kelly M. (2018). Education and Income Predict Future Emotional Well-Being of Whites but Not Blacks: A Ten-Year Cohort. Brain Sci..

[69] Assari S., Caldwell C.H., Mincy R.B. (2018). Maternal Educational Attainment at Birth Promotes Future Self-Rated Health of White but Not Black Youth: A 15-Year Cohort of a National Sample. J. Clin. Med..

[70] Assari S. (2018). Maternal Education and Offspring Educational Success in White and Black Families; Minorities Diminished Returns.

[71] Navarro V. (1989). Race or class, or race and class. Int. J. Health Serv..

[72] Williams D.R., Priest N., Anderson N.B. (2016). Understanding associations among race, socioeconomic status, and health: Patterns and prospects. Health Psychol..

[73] Williams D.R., Sternthal M. (2010). Understanding racial-ethnic disparities in health: Sociological contributions. J. Health Soc. Behav..

[74] Williams D.R. (1999). Race, socioeconomic status, and health. The added effects of racism and discrimination. Ann. N. Y. Acad. Sci..

[75] Oliver M., Shapiro T. (2013). Black Wealth/White Wealth: A New Perspective on Racial Inequality.

[76] Wilson K.B., Thorpe Jr R.J., LaVeist T.A. (2017). Dollar for Dollar: Racial and ethnic inequalities in health and health-related outcomes among persons with very high income. Prev. Med..

[77] Sellers S.L., Neighbors H.W., Zhang R., Jackson J.S. (2012). The impact of goal-striving stress on physical health of white Americans, African Americans, and Caribbean blacks. Ethn. Dis..

[78] Sellers S.L., Neighbors H.W. (1999). Goal-Striving Stress, Social Economic Status, and the Mental Health of Black Americans. Ann. N. Y. Acad. Sci..

[79] Pearson J.A. (2008). Can’t buy me whiteness: New lessons from the Titanic on race, ethnicity, and health. Du Bois Rev..

[80] Valentine C.A. (1968). Culture and Poverty; Critique and Counter-Proposals.

[81] Small M.L., Harding D.J., Lamont M. Reconsidering culture and poverty. Ann. Am. Acad. Polit. Soc. Sci..

[82] Murray C., Herrnstein R. (1994). The Bell Curve: Intelligence and Class Structure in American Life.

[83] James S.A. (1994). John Henryism and the health of African-Americans. Cult. Med. Psychiatry.

[84] Sellers S.L., Neighbors H.W. (2008). Effects of goal-striving stress on the mental health of black Americans. J. Health Soc. Behav..

[85] Bennett G.G., Merritt M.M., Sollers J.J., Edwards C.L., Whitfield K.E., Brandon D.T., Tucker R.D. (2004). Stress, coping, and health outcomes among African-Americans: A review of the John Henryism hypothesis. Psychol. Health.

[86] Thorpe R.J., Kennedy-Hendricks A., Griffith D.M., Bruce M.A., Coa K., Bell C.N., Young J., Bowie J.V., LaVeist T.A. (2015). Race, Social and Environmental Conditions, and Health Behaviors in Men. Fam. Community Health.

[87] South S.J., Deane G.D. (1993). Race and residential mobility: Individual determinants and structural constraints. Soc. Forces.

[88] Bischoff K. (2008). School district fragmentation and racial residential segregation: How do boundaries matter?. Urban Aff. Rev..

[89] Massey D.S., Condran G.A., Denton N.A. (1987). The effect of residential segregation on black social and economic well-being. Soc. Forces.

[90] Brooks-Gunn J., Markman L.B. (2005). The contribution of parenting to ethnic and racial gaps in school readiness. Future Child..

[91] Assari S., Cochran S.D., Mays V. (2018). Income Fails to Protect African American Men from Experiences of Perceived Discrimination While It Does for White Men. Front. Psychol..

[92] Assari S., Lapeyrouse L., Neighbors H. (2018). Income Gradient in Goal Striving Stress: African Americans’ Diminished Return. Medicina.

[93] Wilson F.D., Tienda M., Wu L. (1995). Race and unemployment: Labor market experiences of black and white men, 1968–1988. Work Occup..

[94] Assari S., Caldwell C.H. (2018). Teacher Discrimination Reduces School Performance of African American Youth: Role of Gender. Brain Sci..

[95] Oaxaca R. (1973). Male-female wage differentials in urban labor markets. Int. Econ. Rev..

[96] Altonji J.G., Blank R.M. (1999). Race and gender in the labor market. Handb. Labor Econ..

[97] Reimers C.W. (1983). Labor market discrimination against Hispanic and black men. Rev. Econ. Stat..

[98] Bertrand M., Mullainathan S. (2004). Are Emily and Greg more employable than Lakisha and Jamal? A field experiment on labor market discrimination. Am. Econ. Rev..

[99] Assari S. (2018). Blacks’ Diminished Return of Education Attainment on Subjective Health; Mediating Effect of Income. Brain Sci..

[100] Davis A.J. (1996). Race, cops, and traffic stops. Univ. Miami Law Rev..

[101] Harris D.A. (1996). Driving while black and all other traffic offenses: The Supreme Court and pretextual traffic stops. J. Crim. Law Criminol..

[102] Johnson E.L. (1994). A menace to society: The use of criminal profiles and its effects on black males. Howard L.J..

[103] Assari S., Preiser B., Lankarani M.M., Caldwell C.H. (2018). Subjective Socioeconomic Status Moderates the Association between Discrimination and Depression in African American Youth. Brain Sci..

[104] Assari S. (2017). Combined Racial and Gender Differences in the Long-Term Predictive Role of Education on Depressive Symptoms and Chronic Medical Conditions. J. Racial Ethn. Health Disparities.

[105] Assari S. (2017). Family Income and Depression among Black Youth; Ethnicity by Gender Differences in a National Sample. J. Racial Ethn. Health Disparities.

[106] Hudson D.L., Puterman E., Bibbins-Domingo K., Matthews K.A., Adler N.E. (2013). Race, life course socioeconomic position, racial discrimination, depressive symptoms and self-rated health. Soc. Sci. Med..

[107] Hudson D.L., Neighbors H.W., Geronimus A.T., Jackson J.S. (2012). The relationship between socioeconomic position and depression among a US nationally representative sample of African Americans. Soc. Psychiatry Psychiatr. Epidemiol..

[108] Steele R.E. (1978). Relationship of race, sex, social class, and social mobility to depression in normal adults. J. Soc. Psychol..

[109] Hudson D.L., Bullard K.M., Neighbors H.W., Geronimus A.T., Yang J., Jackson J.S. (2012). Are benefits conferred with greater socioeconomic position undermined by racial discrimination among African American men?. J. Men Health.

[110] Fuller-Rowell T.E., Curtis D.S., Doan S.N., Coe C.L. (2015). Racial disparities in the health benefits of educational attainment: A study of inflammatory trajectories among African American and white adults. Psychosom. Med..

[111] Carbado D.W., Crenshaw K.W., Mays V.M., Tomlinson B. (2013). INTERSECTIONALITY: Mapping the Movements of a Theory. Du Bois Rev..

[112] Mays V.M., Ghavami N. (2018). History, Aspirations, and Transformations of Intersectionality: Focusing on Gender.

[113] Bauer G.R. (2014). Incorporating intersectionality theory into population health research methodology: Challenges and the potential to advance health equity. Soc. Sci. Med..

[114] Bowleg L. (2012). The problem with the phrase women and minorities: Intersectionality—An important theoretical framework for public health. Am. J. Public Health.

[115] Bowleg L. (2008). When Black + lesbian + woman ≠ Black lesbian woman: The methodological challenges of qualitative and quantitative intersectionality research. Sex Roles.

[116] Sheldon K.M., Elliot A.J. (1999). Goal striving, need satisfaction, and longitudinal well-being: The self-concordance model. J. Personal. Soc. Psychol..

[117] Neighbors H.W., Sellers S.L., Zhang R., Jackson J.S. (2011). Goal-striving stress and racial differences in mental health. Race Soc. Probl..

